# Micronutrients and omega-3 PUFAs to promote healthy ageing: informing a physiology-based complementation strategy

**DOI:** 10.1007/s11357-026-02402-9

**Published:** 2026-07-23

**Authors:** Mette M. Berger, Renée Blaauw, Patrizia D’Amelio, Nicole Ehrhart, Adrian F. Gombart, Leah Gramlich, Reto W. Kressig, Philip C. Calder

**Affiliations:** 1 Faculty of Biology & Medicine, University of Lausanne, Lausanne, Switzerland; 2 Division of Human Nutrition, Faculty of Medicine and Health Sciences, Stellenbosch, South Africa; 3 Service of Geriatric Medicine & Geriatric Rehabilitation, Department of Medicine, University of Lausanne Hospital (CHUV), Lausanne, Switzerland; 4 Columbine Health Systems Center for Healthy Aging, Colorado State University, Fort Collins, CO USA; 5 Department of Biochemistry and Biophysics, Linus Pauling Institute, Oregon State University, Corvallis, OR USA; 6 Faculty of Medicine and Dentistry, Department of Medicine, Division of Gastroenterology, University of Alberta, Edmonton, Alberta Canada; 7 Faculty of Medicine, University of Basel, Basel, Switzerland; 8 Faculty of Medicine, University of Southampton, Southampton, UK; 9 NIHR Southampton Biomedical Research Centre, University Hospital Southampton NHS Foundation Trust, Southampton, UK

**Keywords:** Mitochondrial dysfunction, Inflammageing, Immunosenescence, Omega-3 fatty acid, Vitamin, Trace element

## Abstract

Global population ageing is increasing the burden of chronic disease, functional decline, and loss of independence. For most organs, decline in function starts during the 5th decade of life. Micronutrient (MN) insufficiencies/deficiencies are common and may accelerate biological ageing. MNs are required to support multiple physiologic processes, including mitochondrial homeostasis. Age-related declines in organ function are tightly linked to mitochondrial dysfunction, oxidative stress, inflammageing, and immunosenescence. Selected MNs (vitamins B, C, D, E, iron, selenium, zinc) are critical cofactors for mitochondrial function, antioxidant defence, immunity, and resolution of inflammation. Inadequate MN intakes require specific repletion strategies beyond diet for correction. Low intake, (patho)physiological changes, and common medications jointly contribute to compromised MN status. Emerging trials suggest that correcting MN deficiency may restore mitochondrial function and thereby promote healthy ageing. Optimizing intake over the lifespan represents another means to support healthy ageing. Nevertheless, MNs do not work individually but as a web, and may behave differently in different individuals. Further, low status of the n-3 polyunsaturated fatty acids eicosapentaenoic acid (EPA) and docosahexaenoic acid (DHA) is common in older adults, which favours inflammation and functional decline. EPA+DHA work in combination with MNs on inflammation containment. Routine risk assessment, selected biomarker measurement from midlife onwards, and well-designed trials are needed to complement an insufficient status. MN and EPA+DHA optimization should be integral to healthy ageing strategies.

**Supplementary Information:**

The online version contains supplementary material available at https://doi.org/10.1007/s11357-026-02402-9.

## Introduction

**Fig. 1:**
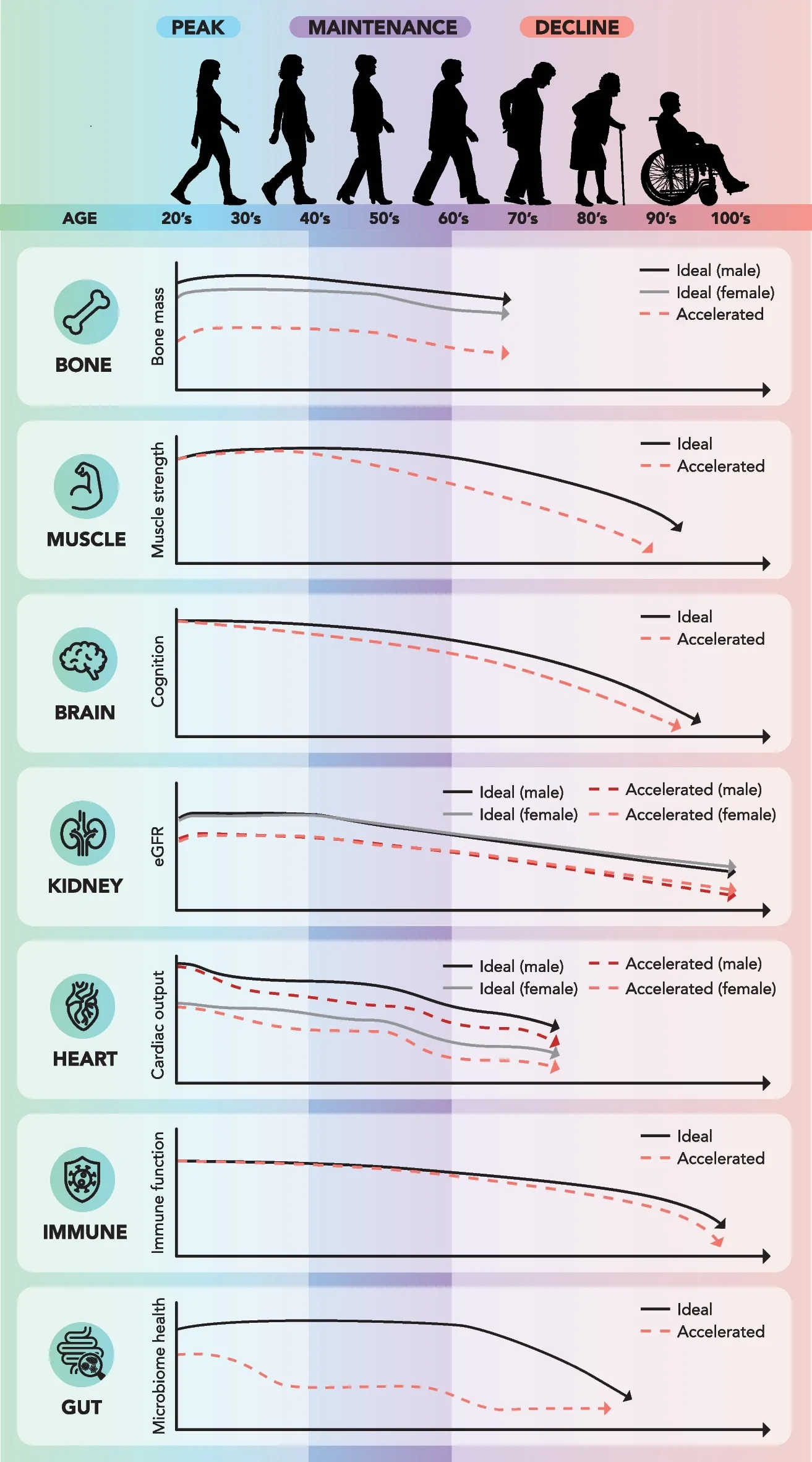
Age-related evolution of function in the major organs, showing that the decline starts during the 5^th^ decade, in the bone [33], muscle [36], brain [37], kidney [38], heart [39], gut microbiome [40], and immune system [41]

Worldwide, the proportion of people aged 65 years and older is rising, reaching 10% of the total population in 2024 [1]. Ageing is often associated with declining function and reduced quality of life. Thus, while longevity (lifespan) has increased, years lived in good health (health span) has not kept pace, making unhealthy ageing a growing societal, medical, and economic concern [2]. Nutrition, which includes micronutrient intake, is an essential health determinant. The term “micronutrient” (MN) refers to vitamins and trace elements [3]. Essential MNs are those the body cannot store enough of to sustain key processes like metabolism, antioxidant defence, immunity, and endocrine activity (Fig. 1). Some MNs are synthesized in the skin (vitamin D) or by the gut microbiota (several B vitamins [4, 5] and K2 [6]), but in insufficient quantities to cover physiological needs. The relationship between diet, health, ageing, and longevity is complex, since unique nutritional components, including both macro- and micronutrients, support many physiologic processes [7]. The World Health Organization (WHO) classifies MN deficiency as a specific form of malnutrition [8] within its 3 categories, highlighting the importance of addressing specific MN needs and optimizing MN intakes to support healthy ageing. The n-3 polyunsaturated fatty acids (PUFAs) eicosapentaenoic acid (EPA) and docosahexaenoic acid (DHA) exhibit MN-like actions, are consumed in mg amounts and intakes, and are typically lower than recommended in older adults [9, 10]; hence, they also warrant consideration in optimization of healthy ageing. Of note, in the 1950 s, polyunsaturated fatty acids (both n-6 and n-3) were referred to as vitamin F [11].

MN deficiencies remain widespread in the global population and require specific strategies beyond diet for correction [12–15]. This manuscript discusses the role of specific MNs and EPA+DHA in promoting healthy ageing of various organ systems. It also addresses specific MN and EPA+DHA needs to prevent unhealthy ageing and improve health span. Malnutrition is now recognized as a key contributor to unhealthy ageing, particularly in accelerating musculoskeletal and cognitive decline [2]. Insufficient intake and deficiency of various MNs may drive, or at least permit, unhealthy ageing. Facing the reality of the frequent MN deficiency [12–15] which negatively affects organ function, the question arises whether MN complements can be recommended? Or should we adhere to the medical teaching that, in general healthy persons, nutritional needs should be met by diet alone? This remains a hot debate after publication of a few negative trials of MNs [16] and editorials warning against waste of money on MN supplements [17], but also arguments about greater likelihood of benefit than harm with MNs and the low cost involved [18]. We propose a physiology-based and individualized approach to complement subjects at risk of deficiency to promote healthy ageing.

## Methods

Authors are experts from different medical disciplines including acute medicine, geriatrics, human nutrition, and veterinary medicine. The expert narrative synthesis was based on a discipline-focused review of the role of MNs and EPA+DHA in physiology, risk modification, and disease prevention, followed by consensus based on epidemiological studies and randomized clinical trials (RCTs), mechanistic work, and observational trials including healthy adults, focused on healthy ageing.

Studies were identified using PubMed, Embase, Cochrane Library, and Web of Science by manual screening of key articles, and through inclusion of landmark publications and those already known to the authors. Time frame was publications 1996 onwards. Given the narrative nature of the review, inclusion was based on relevance to predefined biological and clinical domains and contribution to mechanistic understanding or clinical translation. Priority was given to RCTs, large observational cohorts, and systematic reviews and meta-analyses. Conflicting findings were identified by the authors and discussed qualitatively during online meetings, and email exchanges.

Studies for inclusion in the tables are grouped based on the significance of the effect of the intervention on the primary outcome, i.e. positive, neutral, or negative. Where the effect on the primary outcome was not significant, the study is grouped in the “no effects/neutral” table, irrespective of the findings of effects on the secondary outcomes.

The evidence was contextualized qualitatively, prioritizing consistency across studies, plausibility of biological mechanisms, and strength of clinical evidence.

- **Defining healthy ageing**

Healthy ageing is a dynamic, multi-dimensional process characterized by the preservation of physiological, cognitive, and psychosocial functions that support independence, resilience, and quality of life throughout the lifespan, while minimizing the risk, severity, and duration of chronic disease and disability. As such, healthy ageing is not defined simply as the absence of disease, but by the sustained optimal function of organ systems and cellular processes that underpin health span. The varying sensitivity of different organs to age-related decline further complicates biological ageing, which is highly variable, multifactorial, and non-linear. Recently, epigenetic clocks which mark biological ageing have been used as an outcome in clinical trials [19, 20].

The true prevalence of healthy ageing at the population level remains unknown due to the limited number of cohort studies. A Finnish longitudinal study involving a birth cohort with 13,140 participants reported a probability of healthy survival at age 65 years of 42.8% in men and 40.1% in women. The study found that, among others, nutrition and use of lipid-lowering medications influenced healthy ageing [21].

- **Inflammation and immunosenescence**

Low-grade inflammation increases with age (inflammageing) increasing risk of conditions that contribute to unhealthy ageing [22, 23]. Inflammation is essential for defending against infection, responding to injury and healing wounds. While the goal of inflammation is to damage and kill pathogens, the cellular activities and chemical mediators involved can unintentionally damage host tissues. Fortunately, inflammation is typically self-limiting and resolves rapidly due to endogenous inhibitory processes. One such mechanism involves specialized pro-resolving lipid mediators (SPMs) formed mainly from EPA and DHA which inhibit pro-inflammatory signalling [24]. However, the loss of these anti-inflammatory pro-resolving processes can allow excessive, inappropriate, or chronic inflammation that irreparably damages host tissues leading to pathology and disease, such as rheumatoid arthritis and osteoarthritis, as people age.

Immunosenescence is the name given to age-related immune decline. Inflammageing and immunosenescence are mutually reinforcing: elevated pro-inflammatory mediators in inflammageing suppress adaptive immunity, accelerating immunosenescence; conversely, diminished adaptive immune responses enhance innate immune activity, further promoting inflammation [25]. Mechanistically, this cycle is perpetuated in ageing tissues because compromised mitochondrial biogenesis and quality control result in the accumulation of damaged organelles and excessive production of reactive oxygen species (ROS). These ROS subsequently activate inflammatory signalling pathways, particularly NF-kB, perpetuating a cycle of oxidative damage and inflammation [26]. Together, immunosenescence and inflammageing heighten susceptibility to age-related and infectious diseases, impair vaccine efficacy, delay wound healing, and contribute to increased morbidity and mortality [27, 28].

## Age-related trajectories within body systems

While chronological and biological age may differ, assessing the latter remains challenging, but slowing of telomere attrition has become a widely accepted biomarker [29]. In addition, epigenetic ageing clocks have emerged as promising biomarkers for estimating biological age and for evaluating the effectiveness of healthy ageing interventions [30]. One human study found that higher EPA+DHA status was associated with slower telomere shortening [31], while recent findings from the DO-HEALTH study showed that taking 1 g/day of EPA+DHA for 3 years slowed biological ageing as measured by several epigenetic clocks [19]. Although changes in epigenetic ageing clocks and telomere dynamics provide valuable insights into biological ageing processes, their direct clinical significance in terms of morbidity, mortality, and functional outcomes is still under investigation.

A longitudinal multi-omics study including 108 participants aged 25–75 years identified two key periods of rapid biological change around the average ages of 44 and 60 years [32]. The 40-year transition period is marked by changes in cardiovascular disease risk, and lipid and alcohol metabolism. The transition around age 60 involves changes in immune regulation and carbohydrate metabolism. These findings suggest that the early 40 s may represent a relevant window for initiating “preventive” health interventions. Figure 1 summarizes the evolution of the optimal function of various organs discussed hereafter, and their age-related decline.

- **Bone mass **reaches a peak around age 20–30 years in humans. It remains stable until around age 50 years and then starts to decline [33, 34]. Sex differences exist, as men typically have greater bone mass than women, and women, especially after menopause, experience a more pronounced rate of bone loss [34, 35]. Bone maintenance depends on the interaction of vitamin D, calcium, parathyroid hormone, and insulin-like growth factor [33]. Inadequate diet, unhealthy lifestyle, insufficient physical activity, certain diseases, and some medications can impair acquisition of peak bone mass and accelerate bone loss [33, 34].
- **Muscle mass** and strength peak around age 25 years, after which age-related loss begins; gradually at first, but accelerating with time [36]. By age 80 years, individuals can lose 30–40% of peak muscle mass [35]. A lack of regular physical activity and poor diet with low protein intake accelerate muscle loss [36]. Primary sarcopenia, a muscle disease caused by gradual deterioration of muscle tissue, is a hallmark of ageing, but can develop earlier in life. It is defined by low muscle strength, muscle quantity/quality, and physical performance [42] and is worsened by physical inactivity. After age 60 years, muscle strength declines by 3% per year, and about one in five older adults suffers from functional sarcopenia by age 80 [43]. A decline in the number and function of skeletal stem cells responsible for muscle repair and regeneration occurs with ageing, a phenomenon promoted by inflammation [44].
- **Cognitive function** peaks at 20–30 years of age and starts to decline around age 50–60 years. Age-related decreases in blood flow to the brain, combined with persistent low-grade (neuro)inflammation, contribute to progressive neuronal cell dysfunction and the development of neurological disorders [43, 45]. Insufficient levels of vitamins B6, B9, B12 [46, 47], D [48], K [49], and EPA+DHA are associated with cognitive decline in ageing [50]. In the Framingham study cohort, in 793 individuals who were dementia free at early midlife, higher vitamin D levels were associated with low tau deposition on PET-scan 16 years later [51].
- **Kidney function**: Up to age 60 years, kidney function is similar in women and men, but after that women experience a steeper loss in function [38]. Recently, acute kidney injury has been proposed to represent an impairment of NAD^+^ synthesis from its precursor niacin that can be therapeutically targeted by vitamin B3 [52].
- **Heart and cardiovascular system***:* Cardiovascular disease (CVD) remains the leading cause of global morbidity and mortality, with age being a prominent independent predictor of disease [53]. Delaying cardiovascular ageing may significantly improve the probability of increased health span and longevity [54]. Cardiometabolic disorders including hyperglycaemia, insulin resistance, dyslipidaemia, and arterial hypertension share common pathophysiological mechanisms with ageing [54]. These include a shift in energy substrate utilization with a reduced capacity to oxidize fatty acids, dysregulation of key signalling pathways [53], and mitochondrial dysfunction accompanied by increased oxidative stress [54, 55]. Over time, these age-related changes lead to left ventricular wall thickening, myocardial fibrosis, and diastolic dysfunction. The cellular composition of the cardiovascular system changes with age, resulting in substantial structural and functional remodelling [55] under the influence of inflammation. A significant age-related decline in cardiac output and stroke volume is observed in both sexes [39].
- **Gastrointestinal tract**: Age-related changes include altered taste, smell and gastrointestinal motility, difficulties with chewing and swallowing, and reduced salivary secretion, gastric emptying, biliary function, and pancreatic enzyme secretion [43, 56]. Ageing also affects the enteric nervous system, the gut-associated immune system, and the gut microbiome and may alter MN uptake. Decreased gastric intrinsic factor production with increasing age can impair vitamin B12 absorption.
- **Immune system**: The immune system develops over the first years of life and may decline later in life [41]. There are multiple components contributing to immunosenescence or age-related immune decline [25]. These include reduced output of new immune cells from bone marrow and thymic involution with decreased output of naive T cells. Consequently, the number of T cells in the bloodstream and the ratios of CD4 to CD8 T cells and of naive to memory T cells are all decreased resulting in weakened T cell responses. Furthermore, ageing is associated with impaired function of neutrophils, antigen-presenting cells, and B cells with consequent decreased antibody production and poorer responses to vaccination. The result of this is greater risk and severity of infections, increased hospitalisations, greater use of antibiotics and higher mortality from infections in older people [57].

## Micronutrient needs

International guidelines define MN requirements for the general population as Recommended Dietary Allowances (RDAs) or Dietary Reference Intakes (DRIs) [58]. It is unclear if covering these DRIs/RDAs is only sufficient to prevent deficiency, or is sufficient for optimal organ function or to slow deterioration of organ function and ageing [59]; hence, biomarkers of organ function may better assess MN adequacy. The European Society for Clinical Nutrition and Metabolism (ESPEN) recently published the MN guidelines for clinical nutrition therapy to assist diagnosis and therapy (Suppl. Table 1) [3, 58].

Determining MN status includes taking the patient’s history, making a clinical assessment, and conducting laboratory tests [3]. Biological diagnosis of deficiency primarily relies on blood level measurements; however, cautious interpretation of low blood MN levels is mandatory in the presence of inflammation [60], as many MNs rapidly shift from the bloodstream to tissues, leading to low blood readings [61]. Therefore, low blood levels do not necessarily reflect deficiency or suboptimal status: diagnosis of deficiency requires the presence of either insufficient intakes (+/- clinical signs, symptoms) or objective loss of body fluids, with abnormal low blood levels. Thus, simultaneous measurement of the intensity of inflammation using a surrogate biomarker like C-reactive protein (CRP) improves interpretation of laboratory results, as CRP levels above 10 mg/L may already alter blood levels of most MNs. This is important as low-grade inflammation is common in ageing [62].

Therapeutic use of MNs requires precise wording too. The term “supplementation” is often used too broadly to indicate MN administration, regardless of dosage: “repletion” should be used to describe a restoration of a deficiency to a normal status [3], while “complementation” refers to completing/topping up an intake that does not cover DRI, using nutritional doses.

**Fig. 2:**
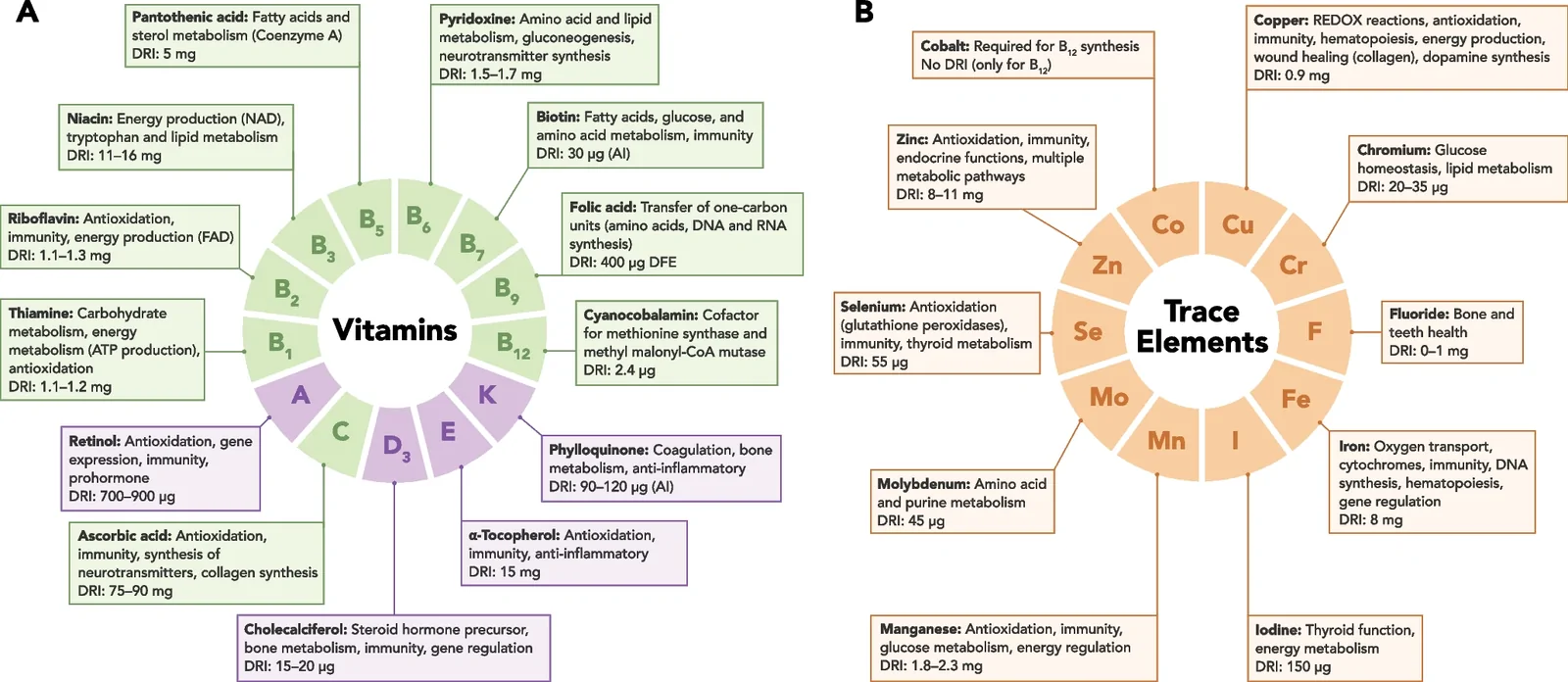
Essential trace elements and vitamins according to the ESPEN-micronutrient guidelines [3]. Only a selection of the predominant functions is reported in a telegraphic style using key words. Water soluble vitamins in green, lipid soluble in violet. Abbreviations: DRI daily dietary reference intakes, AI adequate intake, ESPEN European Society for Clinical Nutrition and Metabolism

While MNs act synergistically, each has specific functions and target sites. Figure 2 summarizes their principal functions. Importantly, several MNs have clear antioxidant functions (Cu, Fe, Mn, Se, Zn, and vitamins A, C, and E) and support immunity [63]. Note: While the minerals calcium, magnesium, and phosphorus are essential, they are not discussed in this review as they do not qualify as trace elements due to their presence in kg proportions in the body, contrasting with MNs (body content of maximum a few mg).

## Consequences of low micronutrient status

- **Mitochondrial dysfunction and oxidative stress**

Mitochondria are the powerhouses of the cell and their abundance in the cytoplasm reflects the metabolic activity of the different tissues and organs [64]. Mitochondrial homeostasis ensures proper energy production and overall cellular health, essential for life maintenance and prevention of disease [65]. Mitochondrial dysfunction, a hallmark of ageing [65], has been associated with suboptimal MN status and impairs cellular energy production resulting in energy deficits with increased oxidative stress and inflammation [66]: ROS result from “electron leak” under normal conditions but production is increased from dysfunctional mitochondria and contribute to increase inflammation.

**Fig. 3:**
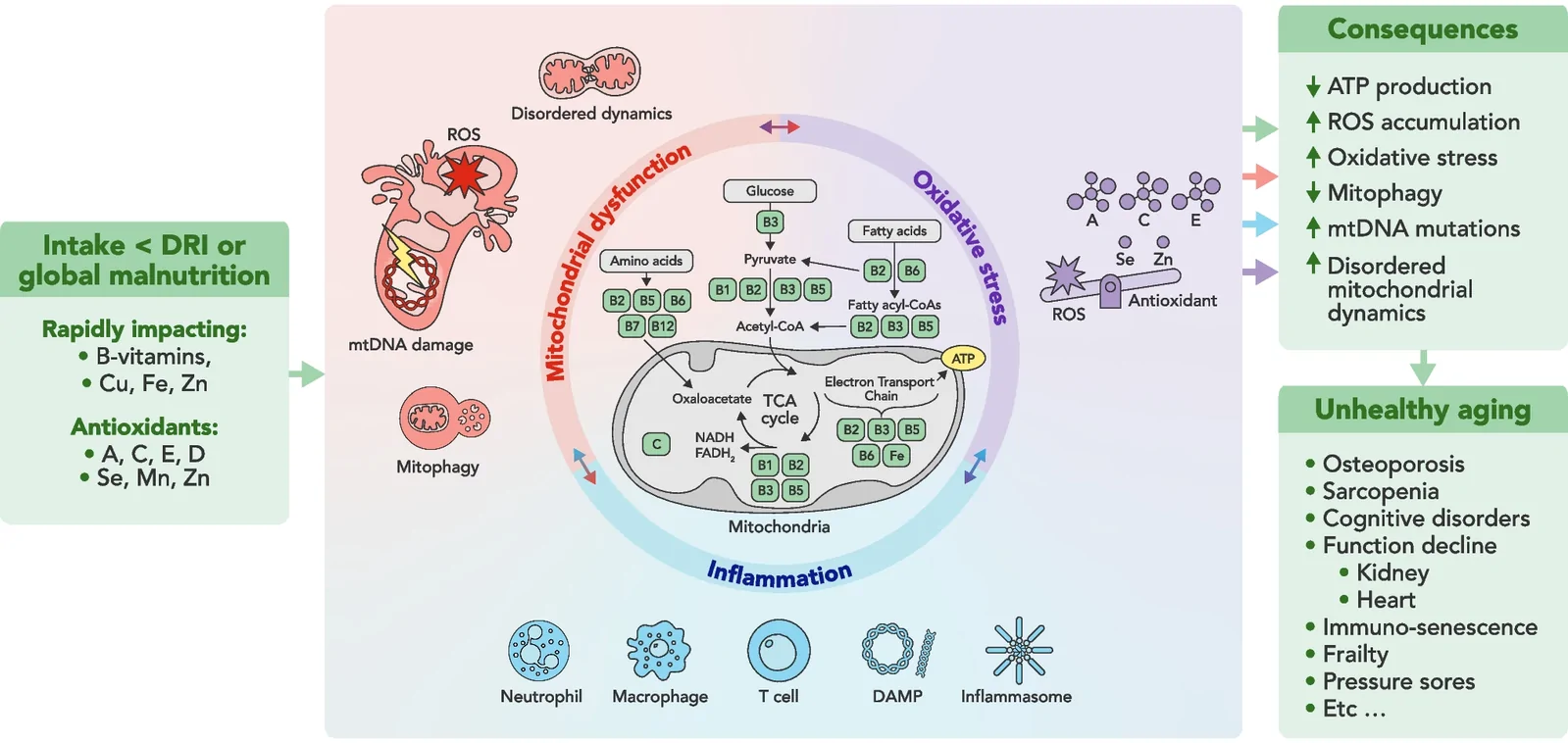
Links between insufficient key micronutrient intakes and progressive mitochondrial dysfunction. Mitochondria convert pyruvate derived from glucose into acetyl-CoA, which enters the tricarboxylic acid cycle (TCA) to produce nicotinamide adenine dinucleotide (NADH) and reduced flavin adenine dinucleotide (FADH2). These molecules transfer electrons to the respiratory chain driving ATP production from ADP through oxidative phosphorylation. During this process, some electrons escape, generating superoxide anion (O_2_^−^), hydrogen peroxide (H_2_O_2_), and other oxidative products. Cells neutralize these highly damaging reactive oxygen species (ROS) with an endogenous antioxidant system comprised of numerous enzymes that require several MN cofactors [65]. Abbreviations: DRI daily dietary reference intakes, Cu copper, Fe iron, Se selenium, Mn manganese, Zn zinc, ATP adenosine triphosphate, CoA coenzyme A, DAMP damage-associated molecular patterns, DNA deoxyribonucleic acid, FADH flavin adenine dinucleotide, NADH nicotinamide adenine dinucleotide, ROS reactive oxygen species, TCA tricarboxylic acid cycle

The different metabolic steps of energy production entirely rely on MNs (Fig. 3). Enzymes of glycolysis and amino acid transamination, of pyruvate and fatty acid conversion to acetyl-coenzyme A and of the TCA cycle, require vitamins B1, B6, B9 (folic acid), and B12. Vitamin B5 is a precursor to coenzyme A, required for pyruvate and fatty acid oxidation, while the electron carriers FAD+ and FMN are synthesized from vitamin B2 and NAD+ and NADP+ from vitamin B3. The cytochrome complexes of the mitochondrial respiratory chain require iron and copper. Hence, generation of energy (ATP) from all fuel sources is entirely dependent upon sufficient MN availability. While these mechanisms are biologically plausible and well supported by experimental data, most human evidence remains observational, and mitochondrial dysfunction in ageing reflects the combined influence of multiple interacting factors beyond MN status alone.

Enzymes involved in mitigating oxidative stress have MNs at their active sites: these include catalase (iron), the superoxide dismutases (copper, zinc, and manganese), and glutathione peroxidase (selenium). Furthermore, vitamins A, C, and E scavenge and detoxify ROS [67, 68] protecting against oxidative stress. Hence, MN deficiency impairs cellular energy production (and many other aspects of metabolism) and promotes oxidative stress, which in turn induces inflammation, partly through production of damage-associated molecular patterns which activate inflammatory cells and upregulate inflammasomes [64]. Hence, mitochondrial dysfunction is a pivotal factor in the progressive age-related decline in cellular homeostasis and organ function [69].

- ➢ **Selenium**: The glutathione peroxidase (GPx) selenoproteins neutralize hydrogen peroxide and lipid peroxides. Low selenium levels are associated with poor muscle strength and mitochondrial abnormalities, including altered cristae structure and reduced respiratory efficiency [70]. Animal studies show that selenium supplementation enhances mitochondrial biogenesis and calcium metabolism, improving muscle performance [71].
- ➢ **Zinc**: As a cofactor for Cu/Zn-superoxide dismutase (SOD1), a key antioxidant enzyme, zinc is essential for mitochondrial redox balance. Observational studies link zinc deficiency, common in older adults, to impaired mitophagy and increased ROS accumulation while studies show that zinc promotes mitochondrial clearance and protects muscle and brain cells from oxidative damage [72, 73]. Zinc also inhibits NF-κB activation in vitro, thereby reducing inflammation [74].
- ➢ **B vitamins**: They directly regulate mitochondrial metabolism [75] and there is evidence from the Mitochondrial Ageing study that deficiencies in vitamins B1 and B6 can compromise mitochondrial bioenergetics, reducing ATP production [76].
- ➢ **Vitamin C**: Among antioxidants that regulate the redox balance in mitochondria, vitamin C maintains physiological ROS-dependent signalling [77].
- ➢ **Vitamin D**: Observational studies link deficiency of vitamin D with reduced oxidative phosphorylation, impaired ATP production, and increased muscle inflammation [78, 79]. Vitamin D receptor signalling modulates the expression of mitochondrial genes and antioxidant enzymes [80]. Vitamin D supplementation potentially restores mitochondrial function and reduces inflammatory cytokine expression in muscle cells [81].
- ➢ **EPA+DHA**: EPA and DHA play a key role in mitochondrial function. In healthy young adult men, daily supplementation with EPA+DHA increased their content in selected phospholipids within skeletal muscle mitochondria, which was associated with enhanced mitochondrial respiration kinetics [82]. In healthy young adult women daily supplementation with EPA+DHA for 4 weeks prior to leg immobilization and then 2 weeks during immobilization mitigated the negative effect of immobilization on skeletal muscle mitochondrial respiration [83]. Furthermore, in obese individuals, daily intake of EPA+DHA for 4 weeks improved peripheral blood immune cell mitochondrial function [84].

Endogenous synthesis of EPA and DHA is low [85]; therefore, dietary sources are essential. EPA and DHA deficiency caused by low intakes is considered one of the major dietary contributors to morbidity and mortality worldwide [86]. EPA and DHA support various cellular and physiological functions, and many cohort studies and RCTs link them to improved health outcomes and reduced disease risk, particularly cardiovascular [87].

- **Attenuated vaccine response**

Consequences of immunosenescence include weakened responses to vaccination and increased risk of infection [57].

- ➢ **Vitamin D**: Low serum vitamin D levels are associated with weaker responses to hepatitis B and possibly COVID-19 vaccines [88–90]. Supplementation in vitamin D–insufficient adults aged 65 years and older enhanced antigen-specific skin responses to varicella zoster virus vaccination [91], but other studies reported no significant improvement in antibody production or seroprotection for influenza or COVID-19 vaccines even in deficient individuals [92, 93]. Still, longitudinal data suggest sufficient vitamin D levels may correlate with higher SARS-CoV-2 antibody titres and reduce post-vaccination seronegativity [94, 95].
- ➢** Iron**: Iron deficiency and low serum iron (hypoferremia) not only cause anaemia but also may impair adaptive immunity and vaccine efficacy. Sufficient iron is essential for optimal production of plasmablasts and IgG responses by human B cells in vitro and in vivo [96]. Globally, iron deficiency has been shown to limit adaptive immunity and responses to vaccines, representing an under-appreciated additional disadvantage to iron-deficient populations [97].
- ➢ **Selenium**: In selenium-marginal adults (blood Se < 110 µg/L), selenium complementation improved T cell proliferation and cytokine production post-influenza vaccination but not flu-specific antibody levels [98]. Complementation of selenium-marginal UK adults enhanced cellular responses and faster viral clearance after oral poliovirus vaccination but did not change antibody production [99].
- ➢ **Zinc**: While one study found no effect of zinc supplementation on influenza vaccine responses [100], others reported improved antibody responses to tetanus and diphtheria vaccines [101]. In healthcare workers, higher free zinc levels were linked to stronger antibody and neutralizing responses to the SARS-CoV-2 vaccine [102].
- ➢ **Combined micronutrients**: A 2-year trial found that daily zinc and selenium—alone or with vitamins—improved influenza antibody titres, while vitamins alone did not [103]. Other small trials using multi-nutrient formulas showed improved responses to specific influenza strains [104], though some found no effect on overall seroconversion [105]. Results varied by supplement type, duration, and vaccine, underscoring the need for further research.

-  **Respiratory infections**

- ➢ **Vitamin C**: Meta-analyses show that regular vitamin C supplementation does not significantly reduce acute respiratory infection (ARI) incidence in the general population [106, 107]. However, vitamin C appears more effective in individuals under physical stress (e.g. athletes, military personnel), where it can substantially lower infection risk [108, 109]. Older adults and those with low baseline vitamin C levels may also benefit, with studies reporting reduced symptom severity and illness duration [110, 111].
- ➢ **Vitamin D**: Several RCTs and meta-analyses suggest that vitamin D provides modest, but statistically significant protection against ARIs, including in older adults [112–114]. However, more recent analyses report inconsistent results [115, 116]. These discrepancies may stem from variations in dosage, background supplement use, and failure to account for baseline vitamin D status. Most meta-analyses rely on study-level data, limiting their ability to detect individual-level effects. Notably, the only individual participant data meta-analysis found a protective effect of vitamin D [114].
- ➢ **Zinc**: In zinc-deficient populations, especially children, supplementation significantly reduces the number of episodes and the duration of acute upper airway infections [117]. In adults, a low zinc status is associated with a reduction of anti- SARS-CoV-2 immunoglobulins A and G [118]. Meta-analysis shows that zinc supplements shorten ARIs [119].

## Factors threatening micronutrient status

- **Inadequate dietary intake**

According to the Determinants of Malnutrition in Aged Persons (DoMAP) model, malnutrition is common in older adults due to reduced intake, lower nutrient bioavailability, and increased requirements [120]. Swallowing and chewing problems, reduced taste, and altered GI motility can exacerbate appetite loss contributing to decreased dietary intake. Comorbidities like GI cancer and disease, neurologic disease, depression/anxiety, frailty, inflammation, and medications can suppress appetite. MNs commonly affected by poor intake include vitamins C, D, E, riboflavin, folate, iodine, iron, zinc, and selenium [15].

Social environment and social ties are essential for maintaining appetite and intake [56]. These factors are central to the longevity observed in Blue Zones, regions found in Japan, Italy, Costa Rica, and Greece, where inhabitants frequently live beyond the age of 100 years [121]. Deceleration of ageing processes in these populations is attributed to predominantly plant-based diets where 90–95% of food comes from vegetables, fruits, beans, nuts, and whole grains, with minimal meat, processed foods, and sugar together with culturally embedded practices, including strong social networks.

Some dietary patterns also correlate with poor MN status. For example, low intake of fruits and vegetables results in lower intake of antioxidant vitamins [122] while strict plant-based diets favour deficiency in vitamins B12 and D, iron, calcium and zinc, and EPA+DHA [123]. Restrictive diets such as low-carbohydrate, vegan, or intermittent fasting invariably limit intake of key MNs [124]. Intake of ultra-processed foods is linked to lower intakes of key MNs with significant deficiencies of vitamins A, C, D, E, B-family vitamins, iron, and zinc [125].

- **Altered gut microbiome**

With ageing, the microbial diversity of the gut microbiome changes, with a gradual decrease in bifidobacteria, which increases the risk of developing cardiometabolic conditions [40, 56]. The microbiome plays a key role in regulation of metabolism and synthesis of some B vitamins [4, 5] and vitamin K2 [6]. Dysbiosis will contribute to low B vitamin status.

- **Use of medications**

Some commonly used medications (Table 1) interact with MN absorption, excretion, and metabolism, with well-defined mechanisms causing deficiency [126, 127]. Databases from the USA and Canada [128, 129] have identified the most frequent drugs used by older people: (1) diuretics (thiazide and loop agents) which cause a direct loss of all water-soluble vitamins (thiamine first line), (2) bile sequestrant drugs and statins which primarily affect fat-soluble vitamins, (3) proton pump inhibitors which compromise the gastric contribution to absorption of vitamin B12, vitamin C, and iron, (4) the antidiabetic metformin, which compromises the status of vitamins B9 and B12, and (5) non-steroidal painkillers which affect the status of vitamins B9 and B12 and iron.

**Table 1:** Drugs impacting micronutrient status (adapted from [126, 127])

Vitamins	Drugs compromising status
Vitamin A	Bile acid sequestrants, statins
Vitamin B1—thiamine	Antibiotics, diuretics
Vitamin B2—riboflavin	Antibiotics, oestrogens
Vitamin B3—niacin	Antibiotics
Vitamin B6—pyridoxin	Antibiotics, anticonvulsants, diuretics, oestrogens, isoniazide
Vitamin B7—biotin	Antibiotics, anticonvulsants
Vitamin B9—folate and folic acid	Antacids, anticonvulsants, bile acid sequestrants, oestrogens, folic acid, metformin, methotrexate, NSAIDs, opioids
Vitamin B12—cobalamin	Antacids, antibiotics, anticonvulsants, oestrogens, H2-receptor antagonists, metformin (biguanides), methotrexate, PPIs
Vitamin C	Antacids, diuretics, oestrogens, PPIs, opioids
Vitamin D	Anticonvulsants, antiretroviral treatments, bile acid sequestrants, corticosteroids
Vitamin E	Bile acid sequestrants, statins
Vitamin K	Antibiotics, vitamin K-dependent anticoagulants, bile acid sequestrants, statins, opioids, corticosteroids
Coenzyme Q10	Statins
**Trace elements**	
Iron	Antacids, bile acid sequestrants, lipid-lowering drugs, NSAIDs, opioids, PPIs
Selenium	Diuretics, statins (interfere with selenoprotein synthesis)
Zinc	Diuretics, oestrogens, lipid-lowering drugs, RAAS inhibitors

*NSAIDs* non-steroidal anti-inflammatory agents, *PPI* proton pump inhibitor, *RAAS* renin-angiotensin-aldosterone-system

Bile sequestrants: colesevelam, colestipol, and cholestyramine (anti-hypercholesterolemia, anti low-density lipoprotein (LDL)). Statins: inhibit enzyme called hydroxymethylglutaryl-Coenzyme A (HMG-CoA) reductase: atorvastatin (Lipitor), simvastatin (FloLipid, Zocor), rosuvastatin (Crestor, Ezallor), cerivastatin (Baycol), fluvastatin (Lescol XL), lovastatin (Altocor, Altoprev, Mevacor, Pravachol), pitavastatin (Livalo, Nikita, Zypitamag), pravastatin (Pravachol)

## Evidence regarding healthy ageing potential of MN complements

Numerous RCTs have been published, with positive results (Table 2), or mixed/neutral, or even negative results (Supplemental Tables 2 and 3).

**Table 2:** Micronutrient studies with positive outcomes (arrows [↓↑] indicate significant changes)

Study author (year)	Population	Intervention	Comparator	Outcomes	Results of intervention compared to control:
Peters et al., 1993 [109]	Ultramarathon athletes (*n* = 68)	Vitamin C (600 mg/d)	Placebo	Upper respiratory-tract (URT) infections	• ↓ Risk of infections (*p* < 0.01) • ↓ Duration of infection (*p* < 0.05)
Girodon et al., 1999 [103]	Adults, aged > 65 years (*n* = 725)	Trace elements: zinc + selenium or vitamins: beta carotene + vitamins C + E for 2 years	Placebo	Immunity	• ↑ Influenza vaccine antibody titres for Trace elements and vitamins combined, or Trace elements alone (*p* < 0.05)
Age-Related Eye Disease Study AREDS Study Group, 2001 [130]	Adults with macular degeneration, aged 55–80 years (*n* = 3640)	Three groups receiving daily supplements for 6.3 years: • Antioxidants: vitamin C (500 mg); vitamin E (400 IU), beta carotene (15 mg) • Zinc (80 mg) and copper (2 mg) • Antioxidants and zinc	Placebo	Age-related macular degeneration (AMD), vision loss	• Combination of antioxidants and zinc resulted in: • ↓ AMD progression (OR 0.72; 99% CI 0.52–0.98, *p* = 0.007) • ↓ Rate of visual acuity loss (OR 0.73; 99% CI 0.54–0.99; *p* = 0.008)
Prasad et al., 2007 [131]	Health adults, aged 55–87 years (*n* = 50)	Zinc (45 mg elemental zinc/d) for 12 months	Placebo	Total infections incidence	• ↓ Infection incidence (29% versus 88%, *p* < 0.01) • ↓ Oxidative stress markers
VITACOG Smith et al., 2010 [146]	Adults, aged ≥ 70 years with mild cognitive impairment (*n* = 271)	Vitamin B6 (20 mg/d) + vitamin B12 (0.5 mg/d) + folate (0.8 mg/d) for 24 months	Placebo	Brain atrophy cognitive decline	• ↓ Brain atrophy rate per year (0.76% versus 1.08%, *p* = 0.001) • More pronounced effects for those with higher baseline homocysteine levels (*p* = 0.001)
VITACOG de Jager et al., 2012 [150]	Adults, aged ≥ 70 years with mild cognitive impairment (*n* = 271)	Vitamin B6 (20 mg/d) + vitamin B12 (0.5 mg/d) + folate (0.8 mg/d) for 24 months	Placebo	Cognitive decline	• ↓ Cognitive decline, especially in those with baseline homocysteine above the median (11.3 mmol/L) • ↑ Global cognition (Mini Mental State Examination, *p* < 0.001) • ↑ Episodic memory (Hopkins Verbal Learning Test–delayed recall, *p* = 0.001) • ↑ Semantic memory (category fluency, *p* = 0.037)
Physicians Health Survey II Gaziano et al., 2012 [132]	Male physicians aged > 50 years (with comorbidities) (*n* = 14,641)	Daily multivitamin for 11.2 years	Placebo	Cancer, Major cardiovascular events	• ↓ Total cancer (HR 0.92; 95% CI 0.86–0.998; *p* = 0.04) • No effect on adverse cardiac events (HR 1.01; 95% CI 0.91–1.10; *p* = 0.91)
CSPPT (China Stroke Primary Prevention Trial) Huo et al., 2015 [133]	Adults with hypertension (*n* = 20′702)	Folic acid (0.8 mg/d) + enalapril for 4.5 years	Enalapril alone	Stroke	• ↓ Risk of first stroke (HR 0.79; 95%CI 0.68–0.93, *p* = 0.003)
COSMOS COcoa Supplement and Multivitamin Outcomes Study Vyas et al., 2022 [137]	Adults, aged > 60 years (*n* = 573 sub-cohort of study)	Cocoa extract (500 mg flavanols/d) and/or a daily multivitamin-mineral (MVM) supplement for 2 years	Placebo	Cognitive change	Participants receiving MVM had: • ↑ Global cognition over 2 y (MD 0.06 SD units; 95% CI −0.003 to 0.13) • ↑ Episodic memory (MD 0.12 SU; 95% CI 0.002 to 0.23) Meta-analysis of COSMOS sub-studies showed MVM benefits: • Global cognition (MD 0.07 SU; 95% CI 0.03 to 0.11; *p* = 0.0009) • Episodic memory (MD 0.06 SU; 95% CI 0.03 to 0.10, *p* = 0.0007) • The magnitude of effect on global cognition was equivalent to reducing cognitive ageing by 2 y
Alehagen et al., 2023 [147]	Adults with low selenium levels (*n* = 441)	Selenium (200 µg/d) + coenzyme Q10 (200 mg/d) for 48 months	Placebo	CVD and age-related markers	• ↓ ICAM-1, adiponectin, SCF, and OPG (*p* ≤ 0.02 for all)
VITAL Telomere trial Zhu et al., 2025 [135]	Adults, aged ≥ 50 years (*n* = 1054)	Vitamin D (2000 IU/d) + EPA+DHA (1 g/d) for 5 years	Placebo	Leucocyte telomere length (LTL) attrition	• Vitamin D3: • ↓ LTL attrition over 4 years (*p* = 0.039) suggesting slower biological ageing • EPA+DHA had no significant effect
DO-HEALTH study Bischoff-Ferrari et al., 2025 [19]	Post hoc analysis on adults, aged ≥ 70 years (*n* = 777)	Vitamin D (2,000 IU/d) and/or EPA+DHA (1 g/d) and/or a home exercise programme over 3 years	Placebo	DNA methylation (DNAm) measures of biological ageing	• EPA+DHA slowed biological ageing over 3 years across three out of four clocks • The magnitude of effect on biological clocks was equivalent to slowing biological ageing by 3 months in 3 years • EPA+DHA, vitamin D, and exercise provided an additive protective effect based on PhenoAge
COSMOS COcoa Supplement and Multivitamin Outcomes Study Li et al., 2026 [20]	Adults, aged > 60 years (*n* = 958 sub-cohort of study)	Cocoa extract (500 mg flavanols/d) and/or a daily multivitamin-mineral (MVM) supplement for 2 years	Placebo	Measures of biological ageing	• MVM ↓ rates of increase of second-generation epigenetic clocks • Cocoa extract had no effect

- **Positive trials**

Thirteen positive RCTs have been published during the last 30 years providing MN combinations at modestly elevated doses (Table 2). The AREDS trial (antioxidants and zinc) showed that zinc reduced progression of macular degeneration and mortality [130]; the zinc trial in older adults showed a reduction of the incidence of infections [131]; the physicians health survey II showed significantly reduced hazard of developing total cancer [132]; the Chinese stroke trial, testing folic acid, showed a significant reduction of stroke [133]; the DO-HEALTH trial which tested combinations of vitamin D, EPA+DHA, and exercise, and initially showed no significant change [134], resulted in slowing of epigenetic methylation clocks by EPA+DHA [19]. The VITAL Telomere trial which tested a combination of vitamin D and EPA+DHA showed a slowing of biological clocks [135, 136]. Finally, the COSMOS trial which tested a widely available inexpensive multi-MN product in older adults, showed significantly better global cognition and episodic memory [137], and slowing of biological clocks [20].

Some RCTs have focused on inflammation. In one study, older women (mean age 72·8 years) received 1 g/day of vitamin C for 6 weeks [138]. While there were no significant changes in leukocyte expression of pro-inflammatory markers, there was a clear trend of decreasing IL-6 and increasing IL-10 mRNA in the ascorbic acid group [138]. Another RCT involving older adults found that a vitamin D analogue increased IL-10 production in LPS-stimulated blood mononuclear cells, although IL-6 induction was unaffected [139]. Extensive research supports the anti-inflammatory actions of EPA+DHA in both model systems and humans [140]. They inhibit pro-inflammatory signalling and the production of pro-inflammatory lipid mediators, while also serving as precursors for anti-inflammatory and pro-resolving mediators [141]. Human trials are discussed in full elsewhere [140]. A comprehensive umbrella meta-analysis of 32 meta-analyses confirmed significant reductions in circulating CRP, IL-6, and TNF-alpha across different population groups [142]. A recent study in Germany tested a combined MN and EPA+DHA intervention in older adults (mean age 75·6 years) [143]: participants received a daily combination of multivitamins including C, D, and K along with zinc, selenium, chromium, molybdenum and iodine, and EPA+DHA for 12 weeks. The intervention significantly reduced a “composite” age-related chronic inflammation score comprised of CRP, white cell counts, platelet counts, and granulocyte to lymphocyte ratio [143]. Thus, one key mechanism by which EPA and DHA support health is through their anti-inflammatory and inflammation resolving actions [141], which likely contribute to healthy ageing in humans and in other species including pets [144, 145].

Some studies have tested “organ specific” MN interventions, not providing the complete blend of MNs. In the VITACOG trial, patients with mild cognitive decline received daily supplements of B (vitamins B6 20 mg, B9 800 µg, and B12 500 µg) for 2 years [47], slowing cognitive decline and reducing brain atrophy by 24%. The greatest benefits were observed in individuals with the highest baseline homocysteine levels (which reflects vitamin B deficiency) [146]. The heart, with its intense continuous activity and high metabolic rate, is highly exposed to oxidative stress. Supporting mitochondrial function with MNs is a cornerstone to maintaining and improving cardiac function [69]. Selenium is an antioxidant candidate confirmed by a Swedish RCT that examined the effects of moderate doses of selenium and coenzyme Q10 over 48 months: five cardiovascular and age-related biomarkers responded favourably, suggesting anti-ageing effects [147].

Note that Iron deficiency anaemia is not addressed herein, even though it is a worldwide public health issue, because it reaches beyond ageing. The treatment of iron deficiency anaemia with intravenous iron in patients with heart failure has been tested in numerous RCTs not reported in Table 2 and shown to reduce cardiovascular events while improving cardiac function [148, 149].

- **Mixed-neutral result trials**

Twenty-five studies are reported in Supplemental Table 2 which achieved mixed results.

Some of the large RCTs included in this group are the study by Hennekens et al. [151] in 22,071 male physicians. Beta-carotene was administered on alternate days for 12 years and had no effect on cancer or CVD incidence. The SELECT trial [152] performed in 35,535 healthy men did not find any effect on prostate cancer using a 4 × 4 approach with vitamin E and selenium. SU.VI.MAX (165) studied 13,017 participants from the general population for 7.5 years with a multivitamin and reported no effect on cancer, CVD or mortality. Sub-group analysis did report a significant decrease for cancer and mortality in men only.

Studies using vitamin D as monotherapy reported neutral results on various outcomes [88, 92, 93, 153–157].

Some further studies were not true intervention trials but used self-reported intake data. In a large US survey of an adult civilian non-institutionalized population (*n* = 21,603), 22.8% reported regular use of supplements, and self-reported better overall health despite no apparent differences in clinically measurable health outcomes [158]. An anonymous questionnaire including 470 participants from different US military units showed that 68% of the participants were using complements, most commonly vitamin, mineral, and protein supplements [159]: military rank, participation status in military operations, and physical activity were the main determinants of the specific supplements used.

The largest null study was reported by Loftfield et al. who aggregated data from 3 prospective US cohort studies based on self-reported supplement use [160]: the AHS (Agricultural Health Study), PLCO (Prostate-Lung-Colorectal), and Ovarian cancer cohort. Among the aggregated 390,124 participants (median age, 61.5 years), 55.4% were male, 40.3% were college educated, and 41.9% were multi-MN users, without a history of cancer or other chronic diseases: 164,762 deaths mainly due to cancer, heart diseases, and cerebrovascular diseases occurred during follow-up. The MV users had no mortality benefit.

- **Negative trials**

Five trials have reported significant negative, deleterious effects of MNs, and contributed to generate a hot debate about the utility and even potential harm of MN interventions [16, 17] (Supplemental Table 3). Among these were the Alpha-Tocopherol Beta Carotene (ATBC) [161] and CARET [162] trials, which tested very high doses of vitamin A and E in a population at higher risk for lung cancer, the SELECT trial which combined high doses of vitamin E and selenium [163], and the HOPE-TOO trial which also tested high doses of vitamin E [164]; doses used in these trials were often above the RDA. These four trials were characterized by excessive doses of vitamin E, a vitamin for which there is a dose response curve in the direction of deleterious prooxidant effect with increasing all-cause mortality as shown by the meta-analysis of Miller et al. [165]. Both the ATBC [154] and CARET [155] trials enrolled subjects who were smokers and provided them with high dose vitamin E supplementation. As cigarette smoking is a well-recognized risk factor for lung cancer and is considered to outweigh all other factors that lead to lung cancer [166], one can argue that the outcome of the studies can be attributed to the smoking history and not the vitamin E supplementation.

Although a large dose of vitamin E (400 IU per day) in the HOPE-TOO trial (157) resulted in increased incidence of heart failure, providing 600 IU per day for 10 years had no effect on CVD and cancer in the Women’s Health Study [167]. In the latter study, healthy women were studied, whereas patients with vascular disease or diabetes mellitus were included in the HOPE-TOO trial. This highlights the importance that not only dosage but also baseline disease condition impact on results.

The 5th study on the table, the Iowa Women’s Health Study, which reported higher risk of mortality, was not an intervention trial [168], but used self-reported long-term intake data.

While these trials have been well conducted and their results are valuable, some study design characteristics may contribute to negative results such as (1) absence of MN dose control with self-reported consumption (no control, no precise recording), (2) addressing already ill populations may be expecting too much, (3) testing a single MN with potential for prooxidant effects at high dose is likely to be deleterious and is discouraged by ESPEN [58], (4) providing an incomplete combination of MNs, and (5) not having considered baseline MN status. Further, in trials, the absence of consideration of the presence of inflammation compromises assessment of baseline MN levels.

An interesting observation is that some trials appear in two different tables, depending on the outcomes reported. COSMOS (2-year duration), DO-HEALTH (3-year duration), and VITAL (5-year duration) have shown positive results when studying measures of biological ageing [19, 20, 135, 137], while they all reported neutral results when studying health outcomes [134, 169, 170]. This highlights the importance of specifying which outcome is referred to when claiming that those studies are positive or neutral. It also shows the importance of allowing adequate time for outcomes to develop and be evaluated, as MNs are not drugs but require metabolic and phenotypic integration time. Another complexity is the type of statistical analysis used can affect the actual findings of RCTs. This is highlighted by the publications from the VITAL trial. The original publication [15] reported little impact of EPA+DHA intervention on cardiovascular outcomes although there was a significant reduction in myocardial infarction. However, a re-analysis of the same dataset came to the conclusion that “daily omega-3 fatty acid supplementation robustly lowers risk of coronary events” and that “probabilities of omega-3 fatty acids being effective were 99.7% for coronary heart disease, 99.6% for total myocardial infarction, 98.4% for cardiovascular disease, 98.8% for all-cause death, 99.8% for cardiovascular death” [136].

## Individualized deficiency correction and complementation strategy

The WHO does not recommend vitamins B and E, EPA+DHA, or multi-MN supplements for prevention of cognitive decline, nor for any ageing prevention purpose. Instead, WHO advises a healthy, balanced diet [171]. The MIND Diet (Mediterranean-DASH diet Intervention for Neurodegenerative Delay) was developed to support brain health during ageing and is indeed associated with better cognitive function [172, 173].

However, based on the reported negative consequences of insufficient or deficient MN intake and status and the numerous positive trials (Table 2), our expert opinion is that covering MN and EPA+DHA needs may prevent, slow or mitigate inflammageing, immunosenescence, and their related diseases especially in individuals with existing deficiencies. The critical MNs to consider include vitamins A, B, C, D, and E, as well as iron, selenium, and zinc, and EPA+DHA [28, 174]. The optimal daily intake of MNs to prevent or slow neurodegeneration and cognitive declines remains unknown [45] (which also applies to other organs) and may be modestly higher than the RDA. Paradoxically excess of antioxidants may negatively impact inflammation by inhibiting immune-suppressing effects of EPA+DHA and by suppressing endogenous antioxidant enzyme synthesis [173]. Based on physiology, multi-MN combinations offer greater potential than individual MNs alone [46].

The objective is to correct suboptimal MN and EPA+DHA levels in individuals at risk for deficiency based on laboratory assessments. A standard check-up at age 50 years could include a panel of blood tests targeting the MNs and omega-3 index [175], focussing on those involved in oxidative stress protection and anti-inflammation (Table 3). Deficiencies require treatment, but suboptimal status may benefit from targeted complementation with MNs known to maintain organ function.

**Table 3:** Proposed list of blood micronutrient analyses to be included in the 50 years’ health screening check-up (first column), and those practically feasible in standard laboratory facilities. Simultaneous assessment of the inflammation level is required (hs-CRP or CRP) also in healthy subjects [3]. This micronutrient list, and at least the minimal short list, should be included in the conventional blood chemistry check-up variables*

Ideally determined MNs [3]	Minimal laboratory outwork
High sensitivity CRP or CRP	CRP
B1—thiamine	Thiamine diphosphate (ThDP)
B2—riboflavin	plasma Flavine Adenine Dinucleotide (FAD)
B3—niacin	-
B6—pyridoxine	whole blood vitamin B6 vitamers
B9—folate	serum Folate #
B12—cobalamin	Cobalamin: combination of holo-transcobalamin [holo-TC], and methylmalonic acid [MMA]
C—ascorbic acid	-
D—25-hydroxy D3	total 25(OH)D [25-hydroxycholecalciferol]
E—α-tocopherol	-
K—phylloquinone	-
Fe—iron	Ferritin, Transferrin
Se—selenium	plasma/serum Selenium
Zn—zinc	plasma/serum Zinc
EPA+DHA: omega-3 index	Erythrocyte omega-3 index (EPA+DHA) [175]

*hs-CRP* high sensitivity C-reactive protein, *CRP* C-reactive protein, *EPA* eicosapentaenoic acid, *DHA* docosahexaenoic acid

#: Folate with B12 together, using a kit with serum or whole blood ; *Variables part of standard check-up: Complete blood count, glucose, electrolytes, HbA1c (haemoglobin A1c), creatinine, total cholesterol, HDL-cholesterol, LDL-cholesterol/apolipoprotein B ratio, alanine aminotransferase (ALAT), aspartate amino transferase (ASAT), TSH (thyroid stimulating hormone)

Correcting suboptimal levels is expected to restore related physiological functions. MN and EPA+DHA intervention may only be effective in cases of baseline deficiency [46]. At very high doses, MNs may have side effects and in some cases may result in toxicity with longer term use [3]. Consultation with the healthcare professional is thus important to prevent accidental overconsumption from different sources. High quality products from reputable manufacturers should be preferred.

From a practical perspective, clinicians should be aware of the high prevalence of MN and EPA+DHA insufficiency in ageing populations and consider targeted evaluation where appropriate, while avoiding indiscriminate supplementation. Importantly, any intervention should be guided by clinical context, baseline status where available, and current guidelines.

Our proposed provocative strategy to introduce a routine biomarker screening around age 50 years faces some challenges. Not the least, there are still no widely available reliable standard MN assays, and there is considerable inter-laboratory variability among some of the tests that are used. The evidence that such screening improves hard clinical outcomes requires large trials, and the cost-effectiveness data to support such a screening programme are missing. The optimal screening time is an “evidence-informed guess” based on the time-point of defining organ functions: it is also not clear how often such screening should be repeated. According to epidemiological [176] and intervention trials [137, 177], every 4–5 years might be sufficient. Healthcare feasibility needs to be addressed and would likely only be present in high income countries. Standardized MN screening is likely to remain largely self-paid in many settings, further limiting access.

## Strengths and limitations

Strengths of this article include the integration of evidence by subject experts and the linking of biological mechanisms, physiological outcomes, and clinical endpoints. However, there are some limitations. The identification of the literature to include was not done systematically and this may introduce selection bias. However, we categorized the included human studies (mainly RCTs) as positive, mixed/neutral, or negative based upon the effect of the MN or omega-3 PUFA intervention on the primary outcome of the study and we fully discussed this literature. We did not include a quantitative synthesis of the studies, maintaining a narrative approach; however, quantitative synthesis would be difficult because of the variable nature of the interventions used and the outcomes reported.

Despite strong biological plausibility and promising preclinical data, MN, antioxidant, and omega-3 fatty acid research has historically struggled to deliver consistent clinical outcomes in human trials. A major hurdle is adapting MN and EPA+DHA trials with good clinical practice as outlined by the International Council for Harmonization (ICH) for pharmaceuticals for human use [178]. RCTs use designs developed for testing drugs, but because MNs, antioxidants, and EPA+DHA are naturally present in the diet of participants, it is not possible to study them in isolation like drugs that are not normally found in study participants. Also, drugs often operate in a linear-dose response manner while nutrients may display U-shaped curve responses [179]. In addition, study design and methodological limitations such as small sample sizes, lack of a well-defined control group, heterogeneous nutrient levels in the populations, variable dosing regimens, short intervention periods relative to disease progression, lack of standardized outcomes or biomarkers for status, compliance issues, and inadequate control for confounders (e.g. inflammation or medication use) make it difficult to interpret intervention effects [180, 181]. Biological complexity and individual variability due to genetic differences, gut microbiome composition, metabolic demands (e.g. obesity vs. malnutrition), and age-related changes likely account for the failure of “one-size-fits-all” supplementation approaches [182]. Targeting high-risk groups rather than general populations may yield more consistent benefits. Measurement issues, the lack of standardized sample handling, and biomarkers for assessing micronutrient status and presence of inflammation further complicate micronutrient and antioxidant/oxidative stress assessment and correlation with clinical outcomes [181]. Finally, confounding actors including comorbidities, medication interactions, socioeconomic status, poor dietary quality, and environmental exposures all influence baseline status and response to supplementation and are often not well controlled for in research designs [183]. Future progress will depend on precision nutrition approaches and rigorous trial design to address current limitations [181, 182].

## Conclusions

The health-promoting biology of MNs and EPA+DHA remains an under-recognized axis of healthy ageing. The evidence reviewed here indicates that suboptimal status of selected vitamins, trace elements, and EPA+DHA is common and may contribute to key biological pathways involved in ageing, including mitochondrial dysfunction, oxidative stress, inflammageing, immunosenescence, and progressive decline across organs and systems. Importantly, these nutrients act within tightly interconnected networks: B vitamins and iron support mitochondrial energy production; vitamins C, D, and E, selenium, zinc, and iron underpin antioxidant and immune defences; and EPA+DHA contribute to the active resolution of inflammation. None of these nutrients operates in isolation, and isolated single nutrient strategies are inefficient and may not bring about the desired improvements.

The emerging findings which link MN and EPA+DHA interventions to slower epigenetic ageing, improved mitochondrial and organ function, and reduced inflammatory burden provide promising support to the hypothesis that optimizing MN and EPA+DHA intake over the lifespan may meaningfully alter biological ageing trajectories. The perspective on multi-MN supplementation should be reconsidered and reoriented towards an individualized approach. At a minimum, clinicians and health systems should recognize MN and EPA+DHA insufficiency as a modifiable determinant of unhealthy ageing, incorporate risk assessment and targeted laboratory evaluation—particularly from midlife onwards—into routine care, and consider rational combinations of MNs and EPA+DHA within safe dose ranges where deficiency or suboptimal status is documented. In parallel, robust long-term trials are needed to define optimal combinations, timing, and doses, and to link these to validated biomarkers of biological ageing and clinically relevant outcomes.

## Supplementary Information

Below is the link to the electronic supplementary material.

ESM 1 Supplementary Material 1 (DOCX 99.5 KB)
