# Supplementary material for: Micronutrients and omega-3 PUFAs to promote healthy ageing: informing a physiology-based complementation strategy
Source: GeroScience. 2026 Jul 23;48(5):6437–61. doi: 10.1007/s11357-026-02402-9 (PMC13601384; doi:10.1007/s11357-026-02402-9)
Supplement: Supplementary file 1 [file 11357_2026_2402_MOESM1_ESM.docx]

**Supplemental Files:**

Tables are intended for publication as an online data supplement

**Supplemental Table 1**: ESPEN definition of depletion, deficiency and therapeutic interventions with micronutrients **[3].**

| **Word** | **Definition** |
| --- | --- |
| **Adequate status** | Absence of any clinical signs or symptoms related to micronutrients, and Blood/plasma concentrations within local reference range |
| **Depletion** | Intake below standard recommendation or Evidence of objective loss of a MN in body fluids compromising status |
| **Deficiency** | Intake below standard recommendation or Evidence of objective loss of a MN in body fluids  AND EITHER: Presence of clinical signs or symptoms, compatible with a micronutrient deficiency  OR Blood/plasma concentrations below reference range together with metabolic effects of inadequacy |
| **Complementation** | Describes the delivery of micronutrients to cover basal needs (DRI) to complete an insufficient intake |
| **Repletion** | Delivery of doses aiming at restoring a normal status in presence of deficiency |
| **Supplement** | Term used when the aim is to deliver higher than standard doses (i.e. significantly superior to DRI) |

**Supplemental Table 2: Micronutrient studies with neutral or mixed outcomes**

| **Study (Year)** | **Population** | **Intervention** | **Comparator** | **Outcomes** | **Results of intervention compared to control:** |
| --- | --- | --- | --- | --- | --- |
| Clark et al., 1996 [184] | Adults  (n=1,312) | Selenium (200 µg/d) for 4.5 years | Placebo | Cancer incidence and mortality | - No effect on basal cell skin cancer (RR=1.10; 95% CI 0.95-1.28) or squamous cell skin cancers (RR=1.14; 95% CI, 0.93-1.39) - ↓ total cancer mortality (RR=0.50; 95% CI 0.31-0.80) - No effect on all-cause mortality (RR=0.83; 95% CI 0.63-1.08) |
| **VECAT Trial**  McNeil et al., 1996 [185] | Adults  (n=1,200) | Vitamin E (500 IU/d) for 4 years | Placebo | Progression rates of age-related cataract | - No effect on incidence of or progression of nuclear, cortical, or posterior subcapsular cataracts |
| Hennekens et al., 1996, [151] | Male physicians, 40-84 years of age  (n=22,071) | Beta carotene (50 mg on alternate days) for 12 years | Placebo | Cancer, CVD | - No effect on malignant neoplasm (RR = 0.98; 95% CI 0.91 -1.06, p=0.65) - No effect on myocardial infarction (RR = 0.96; 95% CI 0.84 -1.09), p=0.50 |
| **SU.VI.MAX**  Hercberg et al., 2004 [176] | General population  (n=13,017) | Daily supplement combining ascorbic acid (120 mg), vitamin E (30 mg), beta carotene (30 mg), selenium (100 µg) and zinc (20 mg) for 7.5 years | Placebo | Cancer, cardiovascular disease, mortality | - No effect on cancer incidence, cardiovascular disease incidence and all-cause mortality - In men $ cancer incidence (RR = 0.69, 95% CI 0.53-0.91, p=0.008) and $ all-cause mortality (RR = 0.63; 95% Ci 0.42-0.93, p=0.02) |
| **RECORD Trial**  Grant et al., 2005 [186] | Elderly fracture patients  (n=5,292) | Secondary prevention  Vitamin D3 (800 IU/d), calcium (1000 mg/d), vitamin D3 (800 IU/d) + calcium (1000 mg/d) | Placebo | Fractures | - No effect (ITT) |
| **Women’s Health Study**  Lee et al., 2005 [167] | Healthy women  (n=39,876) | Vitamin E (600 IU/d) for 10.1 years | Placebo | Cardiovascular events, cancer | - No effect on cardiovascular events (RR = 0.93; 95% CI 0.82-1.05; p=0.26) - No effect on total cancer (RR = 1.01; 95% CI 0.94-1.08; p=0.87) or - No effect on mortality (RR =1.04; 95% CI 0.93-1.16; p=0.53) |
| Rayman et al., 2008 [187] | Adults, aged ≥ 60 years with low selenium status  (n=501) | Selenium (100, 200 or 300 µg/d) for 6 months | Placebo | Thyroid status | No change in thyroid function (p>0.18), despite significant increases in plasma selenium levels in all supplementation groups (p<0.001). |
| **SELECT**  Lippman et al., 2009 [152] | Healthy men  (n=35,533) | Vitamin E (400 IU/d) or  Selenium (200 μg/d) or  Vitamin E (400 IU/d) + selenium (200 μg/d) for 7-12 years | Placebo | Prostate cancer | - No effect on developing prostate cancer:   - Vitamin E (HR=1.13; 99% CI 0.95-1.35; p=0006)   - Selenium (HR=1.04; 99% CI 0.87-1.24; p=0.52)   - Selenium plus vitamin E (HR=1.05; 99% CI 0.88-1.25, p=0.62) |
| **VitaMIND**  Corbett et al., 2012 [154] ashi | Adults, aged ≥ 50 years, with vitamin D deficiency and cognitive impairment  (n=620) | Vitamin D3 (4000 IU/d) for 24 months | Placebo | Cognition | No effect on executive function or cognition. |
| **Physicians’ Health Study II**  Grodstein et al., 2013 [188] | Male physicians, aged ≥ 65 years  (n=5,947) | Daily multivitamin | Placebo | Global composite score averaging 5 tests of global cognition, verbal memory, and category fluency | No effect on mean cognitive change over time |
| Ivory et al., 2017 [98] | Adults, aged 50-64 years with suboptimal selenium status (n=119) | Six groups:  Selenium Methionine in yeast matrix (SeY) at a daily dose of: 0, 50 100 or 200 µ/d; or test meals 3/week with Se-enriched onions (SeO) containing 50 µg/d; or unenriched onions for 12 weeks |  | Immune parameters | - Dose-dependent increase­ in T cell proliferation and cytokine production post-influenza vaccination - No effect on influenza vaccine-specific antibody levels |
| Rizka et al., 2018 [139] | Adults, >60 years of age with various stages of frailty syndrome (n=110) | Vitamin D (alpha-calcidol) 0.5 µg/d for 90 days | Placebo | Inflammatory markers | - ↑ IL-10 (p=0.005) - ↓ IL-6/IL-10 ratio (p=0.008) - No effect on IL-6 (p=0.4) |
| **D2d Trial**  Pittas et al., 2019  [155] | Adults with prediabetes  (n=2,423) | Vitamin D (4000 IU/d) for 2.5 years | Placebo | Diabetes incidence | - No effect on development of type 2 diabetes mellitus (HR=0.88; 95% CI 0.75 -1.04; p=0.12) |
| **VITAL**  Manson et al., (2019) [136, 170] | Men and women aged ≥ 50 years (n=25,871) | Vitamin D (2000 IU/d) + EPA+DHA (1 g/d) for 5.3 years | Placebo | Cancer, CVD | Vitamin D did not affect:   - cancer incidence (HR=0.96; 95% CI 0.88 -1.06; p=0.47) - major cardiovascular events (HR=0.97; 95% CI, 0.85 to 1.12; P=0.69) - cancer mortality (HR=0.83; 95% CI, 0.67 to 1.02 - breast cancer mortality (HR=1.02; 95% CI, 0.79 to 1.31), prostate cancer mortality (HR=0.88; 95% CI, 0.72 to 1.07), colorectal cancer mortality (HR=1.09; 95% CI, 0.73 to 1.62) - myocardial infarction (HR=0.96; 95% CI, 0.78 to 1.19); stroke (HR=0.95; 95% CI, 0.76 to 1.20), cardiovascular mortality (HR=1.11; 95% CI, 0.88 to 1.40) - all cause mortality (HR=0.99; 95% CI, 0.87 to 1.12).   EPA+DHA did not affect:   - major cardiovascular events (HR=0.92; 95% CI, 0.80 to 1.06; p=0.24) - cancer (HR=1.03; 95% CI, 0.93 to 1.13; p=0.56) - stroke (HR=1.04; 95% CI, 0.83 to 1.31) - cardiovascular mortality (HR=0.96; 95% CI, 0.76 to 1.21) - cancer mortality (HR=0.97; 95% CI, 0.79 to 1.20) - all cause mortality (HR=1.02; 95% CI, 0.90 to 1.15).   EPA+DHA:   - ↓ myocardial infarction (HR=0.72; 95% CI, 0.59 to 0.90) - Bayesian reanalyses [136] confirmed robustness of EPA+DHA effects on coronary heart disease and MI (HRs 0.88–0.93 and 0.82–0.90; >99% probability of benefit) with modest effects on cardiovascular disease and cardiovascular and all-cause mortality (HRs~0.91–0.96; ≥98% probability of benefit). |
| Goncalves-Mendes et al., 2019 [92] | Older adults, with Vitamin D deficiency (n=38) | Vitamin D3 (100,000 IU every second day) for 3 months | Placebo | Immune parameters | Higher TGFß plasma levels, but no difference in cathelicidin levels, antibody titres and ROS production. |
| **DO-HEALTH study** Bischoff-Ferrari et al., 2020 [134] | Adults aged ≥ 70 years without major comorbidities  (n=2,157) | Eight groups with combinations of Vit.D (2,000 IU/d) and/or EPA+DHA (1 g/d) and/or a home exercise program for 3 years   - Vit.D3+ EPA+DHA + strength-training exercise program (n = 264); - Vit.D3+ omega-3 (n = 265) - Vit.D3 + exercice (n = 275) - Vit.D3 alone (n= 272) - EPA+DHA + exercice (n = 275); - EPA+DHA alone (n = 269); - Exercise alone (n = 267); | Placebo  (n = 270) | Health outcomes: systolic and diastolic blood pressure (BP), Short Physical Performance Battery (SPPB), Montreal Cognitive Assessment (MoCA), and incidence rates of nonvertebral fractures and infections | - No statistically significant benefits of any intervention individually or in combination for the 6 end points at 3 years. |
| Kashi et al., 2020 [88] | Adults (n=447) | Stimulated sunlight or  Vitamin D3 (1000 IU/d for 4 weeks and 400 IU/day for 8 weeks) | Placebo | Hepatitis B vaccination efficacy | - Low vitamin D status associated with poorer vaccine response - Vitamin D supplementation did not influence response |
| **MVM Users Study** Paranjpe  et al., 2020 [158] | Adults  (n=21,603) | Self-reported use of various vitamin and mineral supplements over 19 years | Non-users | Health outcomes | - Multivitamin users reported 30% better health than non-users (adjusted OR=1.31; 95%CI 1.17 -1.46; p<0.001) - No differences in actual health outcomes (chronic disease, physical distress, or daily activity levels) compared to non-users |
| Fantacone et al, 2020 [189] | Adults, aged 55-75 years (n=42) | Vitamin A (700 μg/day); Vitamin D (400 IU/day); Vitamin E (45 mg/day); Vitamin B6 (6.6 mg/day); Folate (400 μg/day); Vitamin C (1000 mg/day);  Iron (5 mg/day); Copper (0.9 mg/day); Zinc (10 mg/day); Selenium (110 μg/day), for 12 weeks | Placebo | Change in bacterial killing; immune parameters | - No significant effect on :   - Whole blood killing of bacteria   - Neutrophil phagocytosis - ROS production - Significant increase in plasma Vitamin C and zinc levels (p < 0.0001) - ↓ Number of days with reported illness by @70% from 6.4 ± 1.7 in placebo to 2.3 ± 0.8 in treatment arm (p = 0.02 |
| Zychowska et al., 2021 [138] | Women, aged > 65 years (n=24) | Vitamin C (1000 mg/d) for 6 weeks + Training programme | Placebo + Training programme | Inflammatory markers | No effect on IL-1, IL-6, IL-10 and CRP |
| **COSMOS** **COcoa Supplement and Multivitamin Outcomes Study** Sesso et al., 2022 [190] | Adults, aged > 60 years, free from major CVD or recent cancer (n=21 442) | Cocoa extract (500 mg flavanols/d) and/or a daily multivitamin-mineral (MVM) supplement for 3.6 years | Placebo | Cardiovascular disease, cancer, mortality | MVM intervention:   - ↓ lung cancer (HR=0.62; 95% CI: 0.42 – 0.92) - No effect on invasive cancer (HR=0.97; 95% CI: 0.86 - 1.09; p=0.57); prostate cancer (HR=0.82; 95% CI: 0.62 - 1.08) and melanoma (HR=0.86; 95% CI: 0.54 -1.35) - No effect on breast cancer (HR=1.06; 95% CI: 0.79 - 1.42) and colorectal cancer (HR=1.30; 95% CI: 0.80- 2.12) - No effect on composite CVD outcome (HR=0.98; 95% CI 0.88 – 1.12) - No effect on all-cause mortality (HR=0.93; 95% CI 0.81 – 1.08) |
| Kumar et al., 2022 [156] | Adults with major depression and vitamin D deficiency  (n=59) | Vitamin D (60,000 IU every 5 days) for 12 weeks | Placebo | Hamilton Depression scale scores | No effect |
| **D-Health**  Neale et al., 2022 [157] | Adults (unscreened), aged ≥ 60 years  (n=21,315) | Vitamin D3 (60,000 IU per month) for 5 years | Placebo | All-cause mortality | - No effect on:   - all-cause mortality (HR=1.04; 95% CI 0∙93 - 1∙18; p=0∙47)   - cancer mortality (HR=1∙15; 95% CI 0∙96 -1∙39; p=0∙13)   - cardiovascular disease mortality (HR=0∙96; 95% CI 0∙72 - 1∙28; p=0∙77)   - mortality from other causes (HR=0∙83; 95% CI 0∙65 - 1∙07; p=0·15) |
| **CORONAVIT sub-study**  Joliffe et al., 2022 [191] | Adults with sub-optimal vitamin D levels (n=6,200) | Vitamin D (800 or 3200 IU/d) | No vitamin D | Acute respiratory infections; response to SARS-Cov-2 infection | - No effect on the protective efficacy or immunogenicity of SARS-CoV-2 vaccination |
| Ahl et al., 2026 [153] | General adult population  (n=2,434) | Vitamin D (1600 or 3200 IU/d) for 5 years, with a sub-group followed-up for 8 years | Placebo | Incidence of major depressive disorder | Over the 5 year period:   - No effect on depression for those receiving 1600 IU/d (HR=0.78; 95% CI 0.35-1.71, p=0.53) or for those receiving 3200 IU/d (HR=0.57; 95% CI 0.24-1.35, p=0.20)   Over the 7 to 8 year period:   - No effect on depression for those receiving 1600 IU/d (HR=0.61; 95% CI 0.34-1.10, p=0.10) - Trend for ↓ depression for those receiving 3200 IU/d (HR=0.54; 95% CI 0.30-1.00, p=0.05) |

**Supplemental Table 3: Micronutrient studies with negative outcomes**

| **Study (Year)** | **Population** | **Intervention** | **Comparator** | **Outcome(s)** | **Results of intervention compared to control:** |
| --- | --- | --- | --- | --- | --- |
| **ATBC Study** (1994) [161] | Male smokers (Finland)  (n=29,133) | Four groups:  β-carotene (20 mg/d) alone or  vitamin E (50 mg/d) alone or  β-carotene (20 mg/d) and vitamin E (50 mg/d) for 5-8 years | Placebo | Lung cancer incidence | Participants receiving β-carotene alone had:   - ↑ incidence of lung cancer (18%; 95% CI 3-36; p=0.01) - ↑ mortality rate (8%; 95% CI 1-16; p=0.02) |
| **CARET**  Omenn et al., 1996 [162] | Smokers, asbestos-exposed  (n=18,314) | β-carotene (30 mg/d) + vitamin A (25,000 IU/d) for 4 years | Placebo | Lung cancer, mortality | - ↑ incidence of lung cancer (RR=1.28; 95% CI 1.04-1.57; p=0.02) - ↑ mortality (RR=1.17; 95% CI 1.03-1.33; p=0.02) |
| **HOPE-TOO**  Long et al., 2005 [192] | Patients with vascular disease or diabetes mellitus  (N=9,541) | Vitamin E (400 IU/d) for 7 years | Placebo | Cardiovascular events, mortality | - No effect on mortality (p=0.99) - ↑ incidence of heart failure (11 vs 9.6%; RR = 1.17; p=0.02) |
| **SELECT**  Klein et al., 2011 [163] | Healthy men  (n=35,533) | Four groups:  Vitamin E (400 IU/d) alone or  Selenium (200 μg/d) alone or  Vitamin E (400 IU/d) and selenium (200 μg/d) for 7-14 years | Placebo | Prostate cancer | Hazard for developing prostate cancer:   - ↑ with Vitamin E alone (HR=1.17; 99% CI 1.004-1.36; p=0.008) - No effect with Selenium alone (HR=1.09; 99% CI 0.93-1.27; p=0.18) - No effect with Selenium plus vitamin E (HR=1.05; 99% CI 0.89-1.22, p=0.46) |
| **Iowa Women's** **Health study**  Mursu et al., 2011 [168] | Older women  (n= 38,772) | Self-reported use of various vitamin and mineral supplements over 19 years | No supplement | Total mortality | Higher total mortality risk (absolute risk increase):   - Multivitamins: HR=1.06; 95% CI: 1.02-1.10; ARI=2.4% - Vitamin B6: HR=1.10; 95% CI: 1.01-1.21; ARI=4.1% - Folic acid: HR=1.15; 95% CI: 1.0-1.32; ARI=5.9% - Iron: HR=1.10; 95% CI: 1.03-1.17; ARI=3.9% - Magnesium: HR=1.08; 95% CI: 1.01-1.15; ARI=3.6% - Zinc: HR=1.08; 95% CI: 1.01-1.15; ARI=3.0% - Copper: HR=1.45; 95% CI: 1.20-1.75; ARI=18.0% |

*Abbreviations: ITT = Intention to treat; RR = relative risk; OR = odds ration; HR = hazard risk; CI = confidence interval; ARI – absolute risk increase*
